# Synergistic impact of depression and osteoporosis on cardiovascular and all-cause mortality: A cohort study based on NHANES data

**DOI:** 10.1097/MD.0000000000044586

**Published:** 2025-09-19

**Authors:** Xiaoqin Qu, Han Wang, Chao Zhang, Xiaoping Xu, Yong Yi, Qingshan Deng, Jingcheng Jiang

**Affiliations:** a Medical Imaging Center, The Second People’s Hospital of Yibin, Yibin, China; b Clinical Research and Translational Center, Neuroimaging Big Data Research Center, The Second People’s Hospital of Yibin, Yibin, China; c Department of Neurosurgery, The Second People’s Hospital of Yibin, Yibin, China.

**Keywords:** all-cause mortality, cardiovascular disease mortality, depression, NHANES, osteoporosis

## Abstract

Depression and osteoporosis are significant global health issues. Depression affects mental well-being and imposes social and economic burdens, while osteoporosis impacts bone health and raises fracture risk. However, their effects on cardiovascular disease (CVD) mortality and all-cause mortality remain unclear. This study examines their combined impact on cardiovascular and all-cause mortality. We used National Health and Nutrition Examination Survey data from 2005 to 2010, 2013 to 2014, and 2017 to 2018 (n = 19,282; weighted population 155,470,483). Depression was assessed via Patient Health Questionnaire-9, and osteoporosis through dual-energy X-ray absorptiometry scans. Mortality data came from the National Death Index. Participants were categorized into 4 groups based on depression and osteoporosis status. Cox models and Kaplan–Meier analysis were used to assess mortality risk. The primary outcomes were CVD mortality and all-cause mortality. During a median 122-month follow-up, there were 2384 all-cause deaths and 581 CVD deaths. The Dep+/OP+ group had the highest all-cause mortality risk (hazard ratio = 2.80, 95% confidence intervals: 1.92–4.08) and CVD mortality risk (hazard ratio = 2.03, 95% confidence intervals: 1.21–3.39). Compared to the Dep−/OP− group, the Dep−/OP+ and Dep+/OP− groups also had elevated risks. The combination of depression and osteoporosis significantly increases CVD and all-cause mortality risks, particularly in younger individuals and males. These conditions may share biological pathways that increase mortality risk, highlighting the need for integrated clinical management.

## 1. Introduction

Depression, a prevalent mental disorder, continues to demonstrate rising global prevalence and has emerged as a critical public health concern. According to World Health Organization statistics, over 350 million individuals worldwide currently experience depressive disorders.^[1]^ This condition exerts detrimental effects not only on psychological well-being but also imposes substantial social and economic impacts. Empirical evidence indicates that depression has been identified as one of the leading contributors to global disability-adjusted life years and disease burden.^[2]^

Osteoporosis has been recognized as a global public health challenge, affecting approximately 200 million individuals worldwide.^[3]^ The condition’s primary clinical ramifications manifest through necessitating extended rehabilitation periods for affected patients, while concurrently generating substantial healthcare expenditures and intensive caregiving requirements. These compounding factors collectively exacerbate the strain on public health infrastructure.^[4,5]^

Emerging evidence has illuminated the complex interplay between depression and osteoporosis. Depression has been associated not only with reduced bone mineral density (BMD) but also with an elevated risk of fractures.^[6]^ The detrimental skeletal effects extend beyond simple density measurements, as depressive disorders may accelerate bone loss through dysregulation of bone turnover markers.^[7]^ Clinical investigations have identified abnormal levels of these biochemical indicators in depressed populations, with this dysregulation postulated to contribute to osteoporosis pathogenesis.^[7]^ Nevertheless, the clinical implications of their comorbidity on patient prognosis remain incompletely understood. This study investigates potential interactions between depressive symptoms and osteoporotic progression while examining their collective impact on cardiovascular disease (CVD) mortality and all-cause mortality.

In this study, we used data from the National Health and Nutrition Examination Survey (NHANES), a large nationally representative sample, to produce comprehensive results. Our aim was to explore the impact of depression and osteoporosis on CVD mortality and all-cause mortality in adults. The findings may raise awareness among depression patients, osteoporosis patients, healthcare professionals, and policymakers, and promote targeted support to improve patient health.

## 2. Methods

### 2.1. Study population

This study used NHANES data from 2005 to 2010, 2013 to 2014, and 2017 to 2018. NHANES, a survey with a nationally representative sample, offers many advantages. Its data covers a wide range of population characteristics and health indicators. All NHANES protocols were approved by the ethics review committee of the National Center for Health Statistics at the Centers for Disease Control and Prevention, and all participants gave written consent after being fully informed, enabling researchers to conduct diverse health-related studies.^[8]^ Moreover, NHANES includes data on depression, osteoporosis, and mortality,^[9]^ giving researchers a valuable chance to deeply analyze the prevalence of these health issues and their related factors.

### 2.2. Assessment of depression and osteoporosis

Since 2005, NHANES has used the Patient Health Questionnaire-9 (PHQ-9) to assess participants’ depression severity. This 9-item self-rated scale scores each item 0 to 3, with a total of 27. Higher scores mean worse depression. Total scores were calculated from questionnaire data and combined with self-reported or medication use data to strengthen results. A PHQ-9 score of ≥ 10 usually suggests clinically significant depression.^[10]^

The NHANES has implemented standardized dual-energy X-ray absorptiometry scanning protocols through its Mobile Examination Center since 2005, systematically assessing bone health in eligible participants aged ≥ 8 years.^[11]^ All proximal femur scans were conducted using Hologic Discovery series densitometers (Hologic Inc., Bedford), with quality control measures adhering to International Society for Clinical Densitometry guidelines. *T*-scores were calculated using the formula: (measured BMD - young adult mean BMD)/young adult SD, referencing the World Health Organization 1994 diagnostic framework.^[11]^ The *T*-score thresholds used in this study were as follows: *T*-score ≥ −1.0 indicated normal bone density; −2.5 < T-score < −1.0 indicated osteopenia; and T-score ≤ −2.5 indicated osteoporosis.^[12]^ In line with previous epidemiological studies, participants with *T*-scores ≥ −2.5 were classified as non-osteoporotic for the purposes of this analysis.

### 2.3. Determination of mortality data

NHANES acquires mortality and follow-up data via the National Death Index which is maintained by the Centers for Disease Control and Prevention. The NDI encompasses comprehensive death records, including dates, causes, and corresponding International Classification of Diseases, 10th edition codes.^[13]^ NHANES participants are linked to NDI records through the unique identifier SEQN, guaranteeing data accuracy and integrity. Mortality status was updated through April 28, 2022.

### 2.4. Cross-grouping

Participants were stratified into 4 distinct groups according to the presence or absence of depression and osteoporosis:

Dep−/OP−: individuals without depression or osteoporosis;

Dep−/OP+: individuals without depression but with osteoporosis;

Dep+/OP−: individuals with depression but without osteoporosis;

Dep+/OP+: individuals with both depression and osteoporosis.

**Figure 1.**
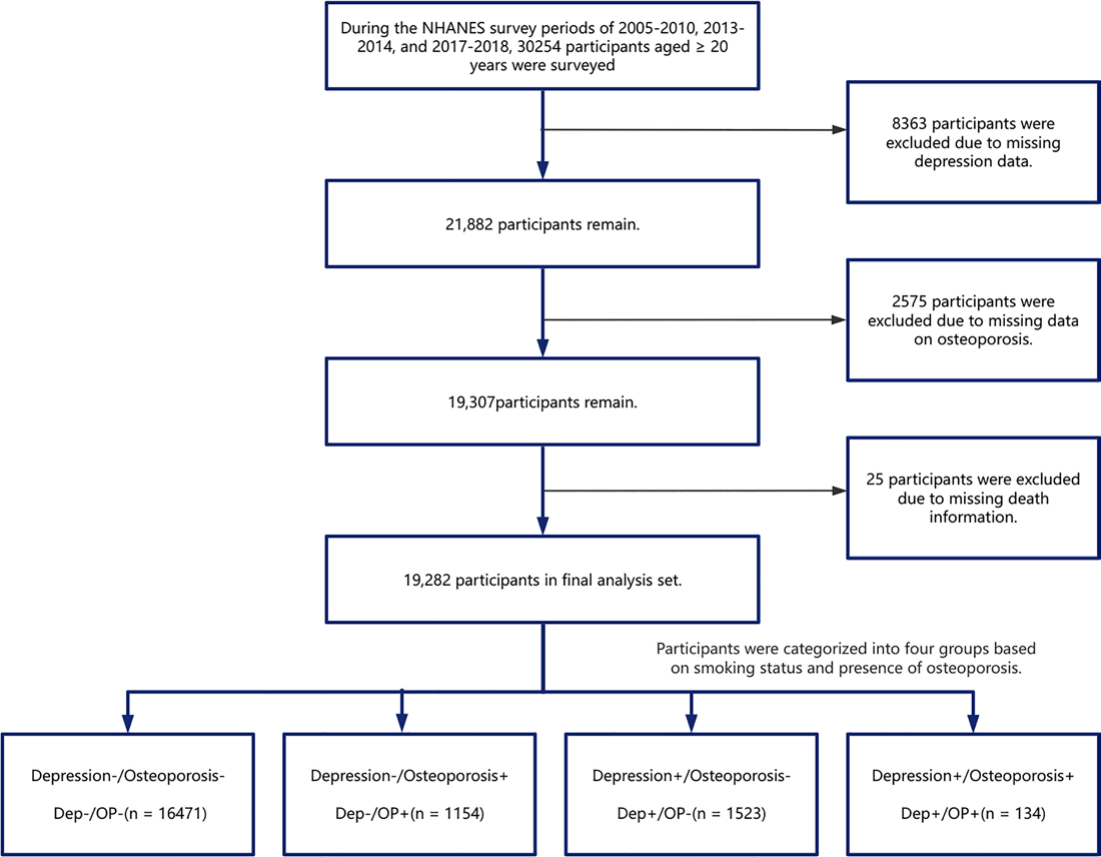
Flowchart for screening and enrollment of study participants.

This cohort study encompassed 19,400 individuals aged 20 years and older. For a detailed illustration of the research process and participant allocation, please refer to Figure 1.

The NCHS Research Ethics Review Committee ensures that informed consent is obtained from all participants. For detailed statistical information, please refer to the website of the National Health and Nutrition Care System (https://wwwn.cdc.gov/nchs/nhanes/default.aspx).

### 2.5. Covariates

In this study, the selection of covariates was guided by previous research.^[14]^ Demographic characteristics included age (continuous), sex (male/female), race/ethnicity (non-Hispanic White, non-Hispanic Black, and other), marital status (cohabiting [married or living with a partner] vs living alone [widowed, divorced, separated, or never married]), family income (categorized by poverty-income ratio), and education level (less than high school vs high school or above). Lifestyle indicators included smoking status (nonsmoker/smoker), drinking status (nondrinker/drinker), and obesity (body mass index [BMI] ≥ 30 kg/m²).

Hypertension (HNT) was defined by any of the following: average systolic blood pressure ≥ 130 mm Hg or diastolic blood pressure ≥ 80 mm Hg (based on the mean of multiple measurements); self-reported history of HNT; current use of antihypertensive medication.^[15]^

Dyslipidemia was identified if any of these conditions were met: serum triglycerides ≥ 150 mg/dL; total cholesterol ≥ 200 mg/dL; low-density lipoprotein cholesterol ≥ 130 mg/dL; high-density lipoprotein cholesterol < 40 mg/dL (men) or < 50 mg/dL (women); undergoing lipid-lowering treatment.^[16]^

Diabetes was determined by any of the following criteria: self-reported clinically diagnosed diabetes; glycated hemoglobin ≥ 6.5%; fasting plasma glucose ≥ 7.0 mmol/L; random plasma glucose ≥ 11.1 mmol/L; 2-hour plasma glucose ≥ 11.1 mmol/L during an oral glucose tolerance test; current use of oral hypoglycemic agents or insulin therapy.^[3]^

### 2.6. Statistical analysis

For descriptive statistics, continuous variables were expressed as means with standard error, while categorical variables were presented as weighted percentages. Group comparisons were conducted using the chi-square test for categorical variables and analysis of variance for continuous variables. Participants were cross-classified participants into 4 groups based on depression and osteoporosis status, and differences among groups compared using the chi-square test. Missing data were addressed using multiple imputation techniques.

The risk of all-cause mortality was assessed using multivariate Cox proportional hazards regression models. Hazard ratios (HR) and corresponding 95% confidence intervals (CI) were computed for the 3 experimental groups (Dep−/OP+, Dep+/OP−, and Dep+/OP+), with the the Dep−/OP− group serving as the reference category. The regression models were adjusted in a stepwise manner: Model 1 was unadjusted; Model 2 was adjusted for demographic factors, including for age, sex, race, marital status, family income, and education level; Model 3 further adjusted for smoking, alcohol use, BMI, HNT, hyperlipidemia (HPL), and diabetes.

Survival analysis was conducted using the Kaplan–Meier method to generate survival curves, and differences among groups were evaluated using the log-rank test. Furthermore, stratified analyses were performed based on age, sex, smoking status, alcohol use, HPL, HNT, and diabetes to assess potential differences among these subgroups.

All the data analyses were conducted using R (version 4.2.2; The R Foundation, Beijing, China) and the Free Statistical Analysis Platform (version 2.1.1; Beijing, China). A 2-sided *P* value of <.05 was considered to indicate statistical significance.

## 3. Results

### 3.1. Baseline demographic characteristics

After excluding 8363 participants with missing PHQ-9 data, 2575 with missing osteoporosis data, and 25 with missing mortality data, a final analytic sample of 19,282 individuals was obtained. The baseline characteristics of excluded and included participants are presented in Table 1. The 19,282 participants (representing a weighted population of 155,470,483) were categorized into 4 groups based on cross-classification. Significant differences in baseline characteristics were observed among the groups (*P* < .001). Specifically, the Dep+/OP+ group exhibited the highest mean age (63.95 years), and the highest prevalence of HNT (61.0%) and diabetes (22.4%). The Dep−/OP+ group had the highest proportion of females (84.8%) and the lowest proportion of participants with a normal BMI (26.0%). The Dep+/OP− group demonstrated the highest rate of alcohol use (69.5%), while the Dep−/OP− group had the lowest mortality rate (8.1%).

**Table 1:** Sample size and participant characteristics of depression symptoms and osteoporosis in the population aged 20 years or older in the National Health and Nutrition Examination Survey of the United States.

Characteristic	Overall, N = 155,470,483	Dep−/OP−, N = 136,017,346	Dep−/OP+, N = 8417,580	Dep+/OP−, N = 10,230,529	Dep+/OP+, N = 805,028	*P*-value*
Age						<.001
Mean (mean std error)	50.55 (0.27)	49.64 (0.27)	65.89 (0.58)	48.95 (0.48)	63.95 (1.31)	
Sex, n (unweighted) (%)						<.001
Male	9804 (49.55%)	8917 (52.29%)	262 (21.25%)	592 (38.42%)	33 (22.80%)	
Female	9478 (50.45%)	7554 (47.71%)	892 (78.75%)	931 (61.58%)	101 (77.20%)	
Race, n (unweighted) (%)						<.001
Non-Hispanic White	9090 (71.25%)	7722 (71.43%)	660 (78.84%)	647 (62.75%)	61 (70.35%)	
Non-Hispanic Black	3867 (10.33%)	3403 (10.36%)	100 (4.11%)	354 (15.47%)	10 (3.71%)	
Other race	6325 (18.42%)	5346 (18.21%)	394 (17.04%)	522 (21.78%)	63 (25.94%)	
Marry, n (unweighted) (%)						<.001
Married	11,903 (66.09%)	10,532 (68.08%)	584 (53.88%)	732 (51.27%)	55 (47.14%)	
Never married	7379 (33.91%)	5939 (31.92%)	570 (46.12%)	791 (48.73%)	79 (52.86%)	
PIR, n (unweighted) (%)						<.001
≤1.30	5589 (18.51%)	4380 (16.60%)	359 (21.51%)	784 (40.52%)	66 (30.58%)	
1.31–3.50	7450 (35.79%)	6366 (35.13%)	508 (44.19%)	520 (36.53%)	56 (49.81%)	
>3.5	6243 (45.70%)	5725 (48.27%)	287 (34.30%)	219 (22.95%)	12 (19.61%)	
Edu, n (unweighted) (%)						<.001
Less than high school	5076 (16.66%)	4075 (15.45%)	347 (20.44%)	586 (28.21%)	68 (34.58%)	
High school or equivalent	4596 (24.42%)	3896 (24.00%)	291 (25.93%)	374 (28.12%)	35 (31.61%)	
Above high school	9610 (58.92%)	8500 (60.55%)	516 (53.63%)	563 (43.66%)	31 (33.81%)	
Smoke, n (unweighted) (%)						<.001
No	10,065 (52.85%)	8736 (53.94%)	673 (55.21%)	599 (37.94%)	57 (32.66%)	
Yes	9217 (47.15%)	7735 (46.06%)	481 (44.79%)	924 (62.06%)	77 (67.34%)	
Drink, n (unweighted) (%)						<.001
No	2601 (10.59%)	2079 (10.02%)	308 (20.95%)	175 (8.81%)	39 (20.28%)	
Yes	16,681 (89.41%)	14,392 (89.98%)	846 (79.05%)	1348 (91.19%)	95 (79.72%)	
BMI, n (unweighted) (%)						<.001
18.5–24.99 kg/m^2^	5466 (30.04%)	4437 (28.69%)	621 (57.27%)	350 (24.21%)	58 (48.41%)	
25.00–29.9 kg/m^2^	6905 (35.20%)	6041 (36.01%)	360 (28.80%)	459 (29.76%)	45 (33.25%)	
≥30.00 kg/m^2^	6911 (34.76%)	5993 (35.30%)	173 (13.93%)	714 (46.03%)	31 (18.34%)	
Hyperlipidemia, n (unweighted) (%)						.004
No	5088 (26.87%)	4467 (27.46%)	244 (22.73%)	353 (23.16%)	24 (18.60%)	
Yes	14,194 (73.13%)	12,004 (72.54%)	910 (77.27%)	1170 (76.84%)	110 (81.40%)	
Hypertension, n (unweighted) (%)						<.001
No	10,419 (59.03%)	9207 (60.77%)	459 (45.35%)	707 (48.63%)	46 (40.67%)	
Yes	8863 (40.97%)	7264 (39.23%)	695 (54.65%)	816 (51.37%)	88 (59.33%)	
DM, n (unweighted) (%)						<.001
No	15,491 (85.27%)	13,352 (85.79%)	918 (84.38%)	1128 (79.57%)	93 (79.85%)	
Yes	3791 (14.73%)	3119 (14.21%)	236 (15.62%)	395 (20.43%)	41 (20.15%)	
All-cause mortality, n (unweighted) (%)	2384 (9.05%)	1801 (7.67%)	352 (26.11%)	185 (11.13%)	46 (36.75%)	<0.001
CVD mortality, n (unweighted) (%)	581 (2.08%)	441 (1.76%)	86 (5.83%)	45 (2.93%)	9 (5.81%)	<.001

CVD = cardiovascular disease.

* Wilcoxon rank-sum test for complex survey samples; chi-squared test with Rao & Scott second-order correction.

### 3.2. Analysis of all-cause mortality risk

**Figure 2.**
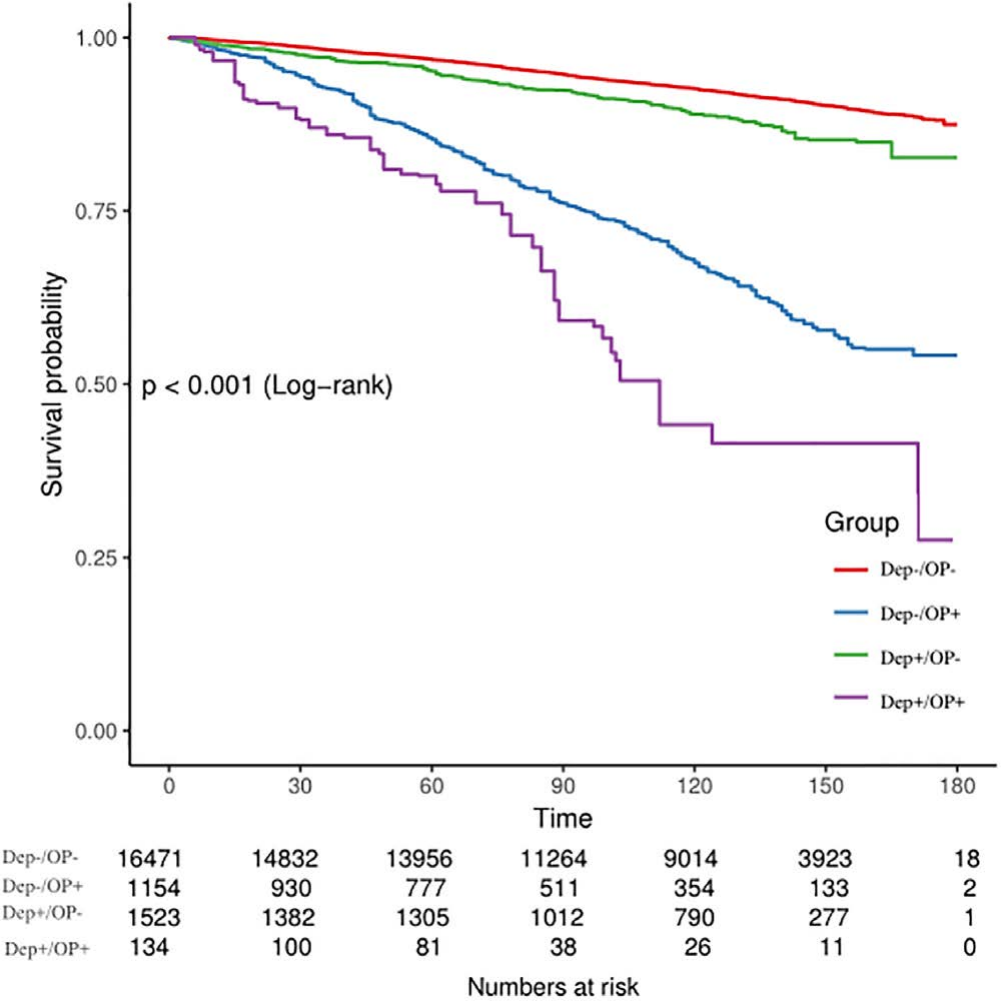
Cumulative survival probability based on cross-grouping of depression and osteoporosis in the US NHANES cohort. NHANES = National Health and Nutrition Examination Survey.

Kaplan–Meier survival curves demonstrated that the Dep+/OP+ group had the lowest survival rate compared with other groups (log-rank *P* < .001, Fig. 2). Cox proportional hazards regression models further elucidated the risk of all-cause mortality. In the fully adjusted Model 3, the Dep+/OP+ group exhibited the highest risk of all-cause mortality (HR = 2.80, 95% CI: 1.92–4.08), followed by the Dep−/OP+ group (HR = 1.69, 95% CI: 1.43–1.99) and the Dep+/OP− group (HR = 1.44, 95% CI: 1.19–1.74). The elevated risk in the Dep+/OP+ group remained statistically significant after adjusting for potential confounding factors (*P* < .001, Table 2).

**Table 2:** Association of depression and osteoporosis with CVD mortality and all-cause mortality among US adults in the National Health and Nutrition Examination Survey.

	Dep−/OP−	Dep−/OP+	Dep+/OP−	Dep+/OP+	*P* trend
All-cause mortality					
Model 1	1.00	5.03 (4.36–5.82) <.001	1.52 (1.26–1.84) <.001	8.92 (6.50–12.22) <.001	<.001
HR (95% CI) *P*-value					
Model 2	1.00	1.69 (1.44–1.99) <.001	1.57 (1.31–1.89) <.001	3.10 (2.11–4.57) <.001	<.001
HR (95% CI) *P*-value					
Model 3	1.00	1.69 (1.43–1.99) <.001	1.44 (1.19–1.74) <.001	2.80 (1.92–4.08) <.001	<.001
HR (95% CI) *P*-value					
CVD mortality					
Model 1	1.00	4.85 (3.84–6.15) <.001	1.75 (1.22–2.50) .002	6.05 (3.20–11.46) <.001	<.001
HR (95% CI) *P*-value					
Model 2	1.00	1.60 (1.25–2.05) <.001	2.01 (1.33–3.02) <.001	2.01 (1.27–3.19) .003	<.001
HR (95% CI) *P*-value					
Model 3	1.00	1.68 (1.30–2.18) <.001	1.86 (1.25–2.77) .002	2.03 (1.21–3.39) .007	<.001
HR (95% CI) *P*-value					

Model 1: unadjusted model.

Model 2: adjusted for age, sex, race, marry, PIR, Edu.

Model 3: adjusted for age, sex, race, marry, PIR, Edu, drink, BMI, hyperlipidemia, hypertension, and DM.

CI = confidence intervals, DM = diabetes mellitus, Edu = education level, PIR = poverty impact ratio.

### 3.3. Analysis of CVD mortality risk

In the Cox proportional hazards regression analysis, after full adjustment in Model 3, the Dep+/OP+ group exhibited the highest risk of CVD mortality with a HR of 2.03 (95% CI: 1.21–3.39). This was followed by the Dep+/OP− group (HR = 1.86, 95% CI: 1.25–2.77) and the Dep−/OP+ group (HR = 1.68, 95% CI: 1.30–2.18). The significantly elevated risk of CVD mortality in the Dep+/OP+ group persisted after adjusting for potential confounding factors (*P* < .001). These results are detailed in Table 2.

### 3.4. Subgroup analysis

**Figure 3.**
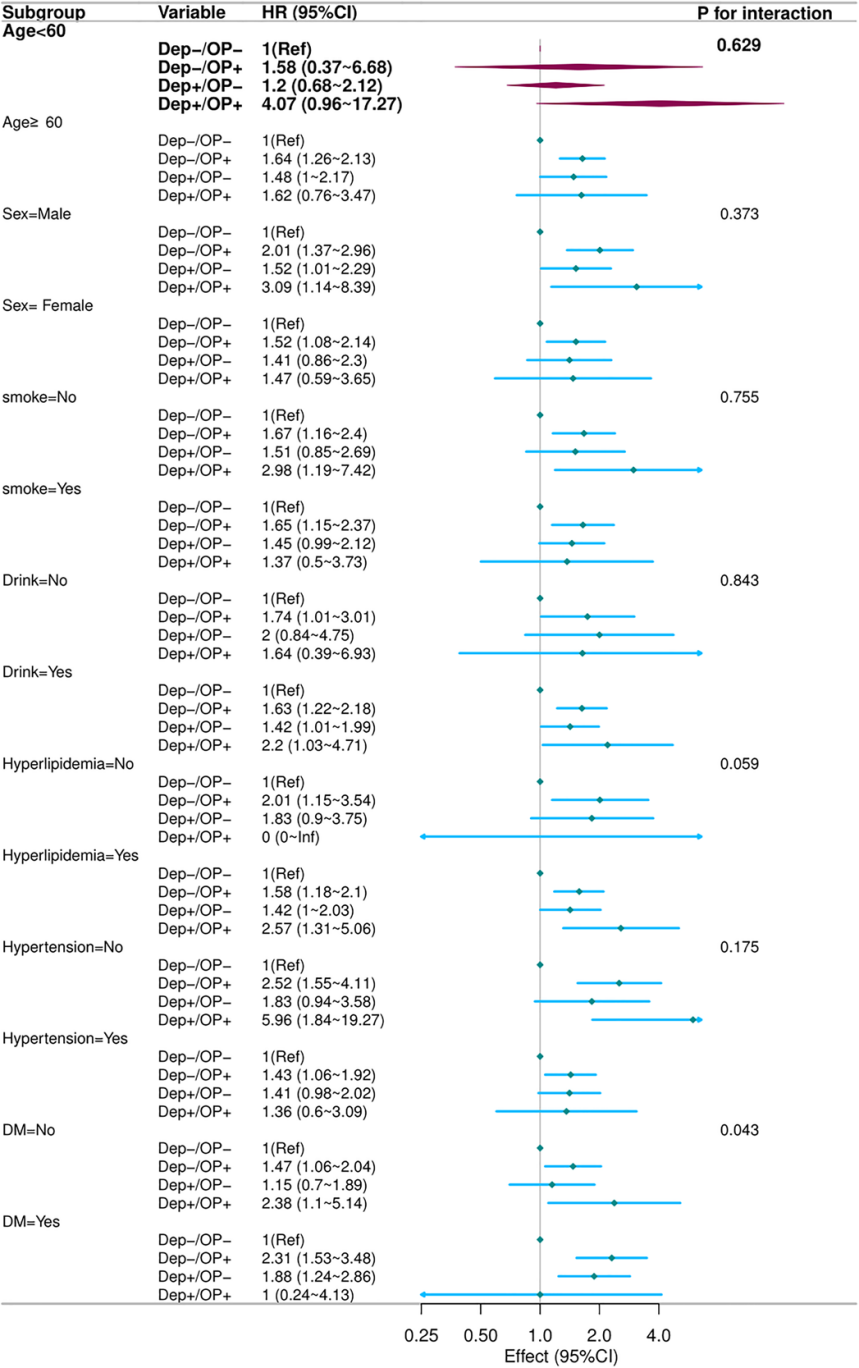
Subgroup analysis of CVD mortality based on cross-grouping of depression and osteoporosis in the US NHANES cohort. The model was adjusted for age, sex, smoking, alcohol use, dyslipidemia, hypertension, and diabetes mellitus. CVD = cardiovascular disease, NHANES = National Health and Nutrition Examination Survey.

**Figure 4.**
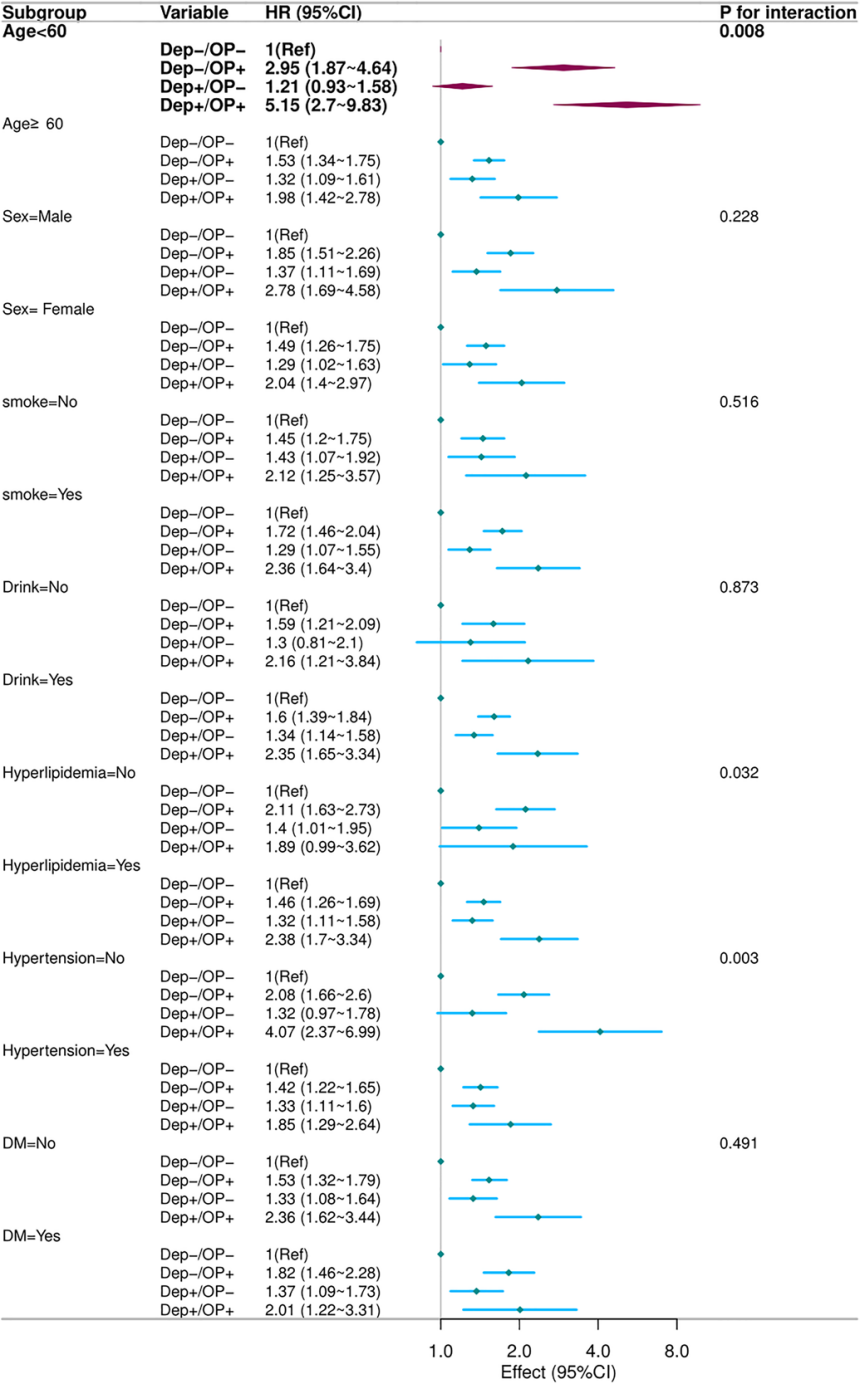
Subgroup analysis of all-cause mortality based on cross-grouping of depression and osteoporosis in the US NHANES cohort. The model was adjusted for age, sex, smoking, alcohol use, dyslipidemia, hypertension, and diabetes mellitus. NHANES = National Health and Nutrition Examination Survey.

Based on the fully adjusted Cox multivariate analysis (Model 3), we performed stratified analyses to further explore the associations. As anticipated, the relationship between depression, osteoporosis, and CVD mortality remained consistent across all subgroups (Fig. 3). Notably, for all-cause mortality, the relationship between smoking, depression, and all-cause mortality was largely consistent across most subgroups. However, significant interaction effects were observed in the stratified analysis of age (*P* = .008), HNT (*P* = .003), and HPL (*P* = .032) (Fig. 4). In individuals younger than 60 years, the all-cause mortality HR for the Dep+/OP+ group reached 5.15, a value significantly higher than the 1.98 observed in those aged 60 years or older (interaction *P* = .008). When HNT was present, the Dep+/OP+ phenotype was associated with an HR of 3.12, exceeding the 2.31 observed in its absence (interaction *P* = .003). Similarly, among participants with comorbid HPL, the HR rose to 3.45, compared with 2.10 in those without HPL (interaction *P* = .032). These findings suggest that the impact of these factors on mortality outcomes may vary among populations with different ages, and in the presence or absence of HNT and HPL.

### 3.5. Sensitivity analysis

To address missing data, we performed multiple imputation for covariates with missing values comprising <10% of the dataset. The consistency of HR before and after re-imputation was excellent, thereby ensuring the stability and robustness of our results.

## 4. Discussion

This large-sample cohort study, utilizing data from the NHANES database, is the first to investigate the combined impact of depression and osteoporosis on CVD mortality and all-cause mortality risks. Multivariable Cox regression analysis revealed that individuals with both depression and osteoporosis (Dep+/OP+) exhibited the highest risk of CVD mortality and all-cause mortality, significantly higher than those with only one of the conditions (Dep+/OP− and Dep−/OP+). Survival analysis further confirmed that the Dep+/OP+ group had the lowest survival rate (log-rank *P* < .001). These findings suggest that the coexistence of depression and osteoporosis may synergistically increase mortality risk through shared biological pathways like chronic inflammation and oxidative stress. Subgroup analysis revealed that the Dep+/OP+ risk was particularly pronounced in younger people (age < 60 years, HR = 5.15 vs older group HR = 1.98, *P* = .008) and higher in males than females (HR = 2.78 vs 2.04), indicating age and sex may modify their combined effect of these 2 conditions on mortality.

The association between depression, osteoporosis, and CVD mortality has been well-established in numerous studies. Research consistently demonstrates that individuals with depression exhibit significantly higher mortality rates compared to those without depression, particularly among males.^[17]^ Depression exerts a profound impact on both mental and physical health through various mechanisms, including behavioral, physiological, and biochemical pathways. A robust link between depression and CVD, with depression recognized as an independent risk factor for CVD.^[18]^ Additionally, osteoporosis is associated with an increased risk of CVD, especially among the elderly.^[19,20,21]^ As an independent risk factor, osteoporosis interacts with depression in complex ways. Studies have found that depression are more susceptible to osteoporosis due to lifestyle changes, such as reduced physical activity and poor diet, as well as the direct impact of depression on bone metabolism.^[6]^ The lower bone density and higher fracture risk in individuals with depression further exacerbate all-cause mortality risk.^[22,21]^ Moreover, osteoporosis can can lead to depression due to declines in physical function and quality of life,^[23,22]^ creating a vicious cycle that further increases mortality risk. Our study identified a significantly elevated risk in younger people, which contrasts with most existing research suggesting that older individuals are more affected by multimorbidity due to higher disease burden.^[23,24]^ This discrepancy may be attributed to the faster decline in bone density in younger individuals, as well as the and earlier accumulation of behavioral risks factors associated with depression, such as smoking and alcohol use.^[25]^ Excessive hypothalamic–pituitary–adrenal (HPA) axis activity in patients with major depressive disorder is typically manifested by elevated cortisol secretion, which in turn disrupts bone metabolism through multiple pathways.^[26]^ Sustained activation of the HPA axis may further accelerate bone loss by altering neuroendocrine and immune signaling.^[27]^ Importantly, pharmacological treatment of depression can partially restore skeletal homeostasis, an effect that appears to be mediated, at least in part, by normalization of HPA-axis function.^[28]^

This strengths of this study are notable. First, we utilized the NHANES database with includes 19,282 nationally representative samples, thereby enhancing the generalizability of our findings. Second, we employed rigorous statistical methods. Specifically, weighted multivariable Cox proportional hazardsmodels were used to adjust for a comprehensive set of confounders, including demographic, behavioral, and metabolic confounders. Additionally, multiple imputation was performed to address missing data, ensuring the robustness and reliability of our results. Finally, subgroup analyses revealed significant modifying effects of age and sex on the associations under investigation, providing valuable insights for targeted and precise interventions in specific populations.

The limitations of this study should be acknowledged. First, we did not include data on fracture history, bone metabolism markers (e.g., CTX, P1NP), or the use of anti-osteoporosis medications, The absence of these variables may have led to an underestimation the biological mechanisms underlying the associations between depression, osteoporosis, and mortality. Second, the assessment of depression was based on questionnaires or self-reports, which may be subject to misclassification bias.

Future research endeavors will focus on integrating bone turnover markers, inflammatory factors (e.g., IL-6, TNF-α), and genetic data to elucidate the biological pathways linking depression, osteoporosis, and mortality. Additionally, leveraging the insights from our subgroup analyses, we plan to develop, age, and sex-specific risk prediction models. These models will serve as valuable tools to guide screening efforts targeting high-risk populations, thereby enhancing the precision and effectiveness of preventive interventions.

This study demonstrates that the comorbidity of depression and osteoporosis significantly amplifies the risks of CVD and all-cause mortality risks, with particularly pronounced effects observed in younger individuals and males. The synergistic impact of these conditions underscores the necessity for clinical practice to adopt an integrated approach to managing mental and bone health. Early intervention strategies for patients with comorbid depression and osteoporosis, such as psychological support, bone density monitoring, and lifestyle modifications, should be prioritized. Further mechanistic studies and intervention studies are warranted to validate these associations and translate the findings into evidence-based clinical practice guidelines.

## Author contributions

**Conceptualization:** Jingcheng Jiang, Xiaoqin Qu, Chao Zhang, Yong Yi, Qingshan Deng.

**Data curation:** Jingcheng Jiang, Xiaoqin Qu, Han Wang, Chao Zhang, Yong Yi.

**Formal analysis:** Jingcheng Jiang, Xiaoqin Qu, Han Wang.

**Funding acquisition:** Jingcheng Jiang, Han Wang, Xiaoping Xu.

**Investigation:** Jingcheng Jiang, Han Wang, Xiaoping Xu.

**Methodology:** Jingcheng Jiang, Han Wang.

**Project administration:** Jingcheng Jiang, Han Wang, Chao Zhang.

**Resources:** Jingcheng Jiang, Xiaoping Xu, Yong Yi.

**Software:** Jingcheng Jiang, Xiaoping Xu.

**Supervision:** Jingcheng Jiang, Yong Yi, Qingshan Deng.

**Validation:** Jingcheng Jiang, Xiaoping Xu, Qingshan Deng.

**Visualization:** Jingcheng Jiang.

**Writing – original draft:** Jingcheng Jiang, Xiaoqin Qu, Qingshan Deng.
